# The Effect of Bisphosphonates on Fracture Healing Time and Changes in Bone Mass Density: A Meta-Analysis

**DOI:** 10.3389/fendo.2021.688269

**Published:** 2021-08-30

**Authors:** Yongquan Gao, Xiaochen Liu, Yuan Gu, Deye Song, Muliang Ding, Lele Liao, Junjie Wang, Jiangdong Ni, Guangxu He

**Affiliations:** ^1^Department of Orthopedics, The Second Xiangya Hospital, Central South University, Changsha, China; ^2^Department Radiology, University of Toledo Medical Center, Toledo, OH, United States

**Keywords:** bisphosphonates, fracture healing, bone mass density, bone turnover markers, meta-analysis

## Abstract

**Background:**

Osteoporosis is a common complication of acute fracture, which can lead to fracture delayed union or other complications and resulting in poor fracture healing. Bisphosphate is a common anti-osteoporosis drug, but its application in fracture patients is still controversial because of its inhibitory effect on bone resorption.

**Method:**

Studies were acquired from literature databases in accordance with established inclusion criteria. Standard mean difference (SMD) and 95% confidence intervals (Cls) were calculated to evaluate the effectiveness of the bisphosphonates treatment in fracture patients. Data analysis was conducted with the Review Manager 5.4.1 software.

**Results:**

A total of 16 studies involving 5022 patients obtained from selected databases were examined. As expected, bisphosphate had no significant effect on fracture healing time, but it could significantly increase BMD and prevent osteoporosis. Meanwhile, bisphosphate can inhibit both bone resorption and bone formation markers, resulting in low bone turnover state.

**Conclusion:**

This meta-analysis showed that bisphosphonate have no significant effect on fracture healing time but they do increase the changes in BMD and reduce bone synthesis and resorption markers. Early application of bisphosphonates after injury in the appropriate patient population should be considered.

## Introduction

Osteoporosis is a common orthopedic process that increases the incidence of pathologic fractures. There are 8.9 million osteoporotic fractures per year worldwide (1). Disuse osteopenia of the affected extremity can occur within a few months of acute fractures. Most significant bone density loss is often found in the hips and vertebral bodies. Such bone density loss can result in osteoporosis in patients with pre-existing osteopenia or risk factors. Standard treatment for fractures include reduction, splinting, external fixator, and plate/screw fixation. Anticoagulation and nonsteroidal anti-inflammatory drugs (NSAIDs) are supplemented to prevent thromboembolic events and pain control.

Bisphosphonate (BP) prevent bone mass loss by inhibiting osteoclast resorption. While it is an effective anti-osteoporotic and is widely used in the world, it is limited by its side effects (2, 3) and is thus reserved for patients with pre-existing conditions including osteoporosis, metastatic bone disease, multiple myeloma, Paget’s disease, polyostotic fibrous dysplasia, total joint arthroplasty, early stage avascular necrosis, osteogenesis imperfecta, metastatic hypercalcemia (4). Recent studies have shown that due to its inhibitory effect on osteoclasts (5), BPs can down-regulate bone metabolism (6, 7), which can lead to a low bone turnover state. Medication compliance is also an issue and the noncompliance rate increases with length of use (8) and the rate of compliance decreases with time. A study of primary physicians, anti-osteoporosis treatments is more likely to be overlooked than treatment for cardiovascular diseases (9).

For fracture patients, the use of BPs can significantly reduce the recurrence rate of fractures (10). However, the role of BPs in fragility fracture healing is not well elucidated with study results ranging from improved healing, no effect, and inhibition of healing (4, 11–17). Earlier studies have shown that the incidence of low impact hip fractures and the rate of recurrent fractures is significantly increased after more than 5 years of long-term bisphosphonate treatment. However, some patients in the above studies are not simply osteoporotic patients. For instance, patient may also have Paget’s disease, which could have influenced the clinical outcome. In animal studies, BPs have been shown to cause low bone turnover (18). Other studies have shown that bisphosphonate can promote fracture healing *in vivo* and *in vitro* (14, 19), accelerating the formation of trabecular bone and the thickness of cortical bone callus (20). There is a lack of sufficient data and human research on the effect of BPs on fracture healing.

The main purpose of this article is to explore the effect of BPs on fracture healing. The main parameters include bone mineral density (BMD) changes, healing time, and bone metabolism indicators. The goal is to clarify if BPs can be used to promote fracture healing after fractures.

## Method

Meta-analysis was performed following Preferred Reporting Items for Meta-Analyses(PRISMA) criteria.

### Search Strategy

This meta analysis was conducted according to the Preferred Reporting Items for Meta-Analyses statement (21). PubMed was searched in June 2021 for studies published between 1987 (first used in clinical treatment) and June 2021 using the following combination of terms: (((((((((((Bisphosphate[Title]) OR (Alendronate[MeSH Terms])) OR (Zoledronic acid[MeSH Terms])) OR (neridronate[MeSH Terms])) OR (Olpadronic acid[MeSH Terms])) OR (Risedronic Acid[MeSH Terms])) OR (Ibandronic Acid[MeSH Terms])) OR (Clodronic Acid[MeSH Terms])) OR (Pamidronate[MeSH Terms])) OR (tiludronic acid[MeSH Terms])) OR (Etidronic Acid[MeSH Terms])) AND (((fracture healing[MeSH Terms]) OR (bone remodeling[MeSH Terms])) OR (fracture[MeSH Terms])). No language restrictions were applied. Two investigators (Yongquan Gao and Yuan Gu) independently completed the search and assessed the identified titles for relevance. Abstracts were screened for all potentially relevant titles, and full papers were obtained for all abstracts of potential relevance. In addition, for trials with several treatment groups, the eligibility of each individual group was assessed and only those relevant were included. The reference lists of the selected papers were also screened for articles that may have been overlooked in the initial search, and references cited in the identified articles were searched manually.

### Selection Criteria

This meta-analysis followed a detailed, pre-specified protocol that set out the objectives, inclusion criteria for trials, data to be collected, and analyses to be completed.

Studies were considered for inclusion if they met the following criteria: (1) A controlled trial of BPs and placebo in the study; (2) participants were adults with acute fractures and were accepting BP therapy following surgical repair or manual reduction of the fracture; (3) the intervention was the initiation of BPs earlier than 3 months compared with the initiation of placebo at the same time, BPs begun later than 3 months after surgery, or no therapy; (4) trials provided the relevant data.

Studies were considered for exclusion if they met the following criteria: (1) participants previously used BPs or parathyroid hormone, unless patients had undergone a washout period; (2) participants involved in tumor, Paget`s disease, pregnancy, dialysis, organ transplantation, secondary fracture or other diseases that may affect bone healing; (3) the fracture treatment involved inserting prostheses, such as total hip arthroplasty (THA); (4) If multiple studies from the same series were available, the one including the most individuals was used in the analysis.

### Data Extraction and Quality Assessment

The following information from the article and the details of group allocation were reviewed: first author, year of publication, age, gender, type of fracture and treatment, type of Bisphosphate, length of BP used, and the body mass index (BMI) and bone mineral density (BMD). All data were thoroughly checked for consistency, plausibility, and integrity of randomization and follow-up. The two responsible trial investigators resolved any queries and verified the final database entries. The primary outcome was the time of fracture healing, BMD changes and bone metabolism biomarkers. Fracture healing is defined as fracture bridging by trabeculae or osseous bone in at least one cortex as seen on anteroposterior radiographs and one as seen on lateral radiographs, The above process requires at least 2 orthopedic or radiologists obtaining same diagnosis under double blindness. Part of the data is obtained by analyzing Figure from the full text, using GetData Graph Digitizer (version2.26.0.20).

The quality of evidence of outcomes was judged according to the Cochrane Scale (22). Cochrane Scale scores ranges from 0 to 7, with higher scores indicating better quality.

### Statistical Analysis

Data were analyzed using Review Manager Software (RevMan version 5.4.1; The Nordic Cochrane Center, The Cochrane Collaboration, Copenhagen, Denmark). The SMD in continuous outcomes and risk ratios (RR) in dichotomous variables with 95% CI and P values were calculated to assess effects of study. In meta-analysis, SMD is applied as an aggregate statistics when all trials evaluated the same outcome, but assessed it with many kind of methods (i.e. different rating scales) (23). We used the inverse variance method in continuous variables with random effects model and/or fixed effects model to combine data and generate the overall effect estimate according the degree of heterogeneity. The degree of heterogeneity was assessed by a χ2 test combined with the I^2^ method (I^2^ < 25% representing low heterogeneity, and I^2^ > 75% representing high heterogeneity) (24). High heterogeneity is modeled with random effects, and vice versa with fixed effect models. The analysis was performed with Revman version 5.4.1. P < 0.05 represents statistically significant. Funnel plotting were used to assess publication bias with Revman version 5.4.1.

## Result

### Literature Selection

**Figure 1:**
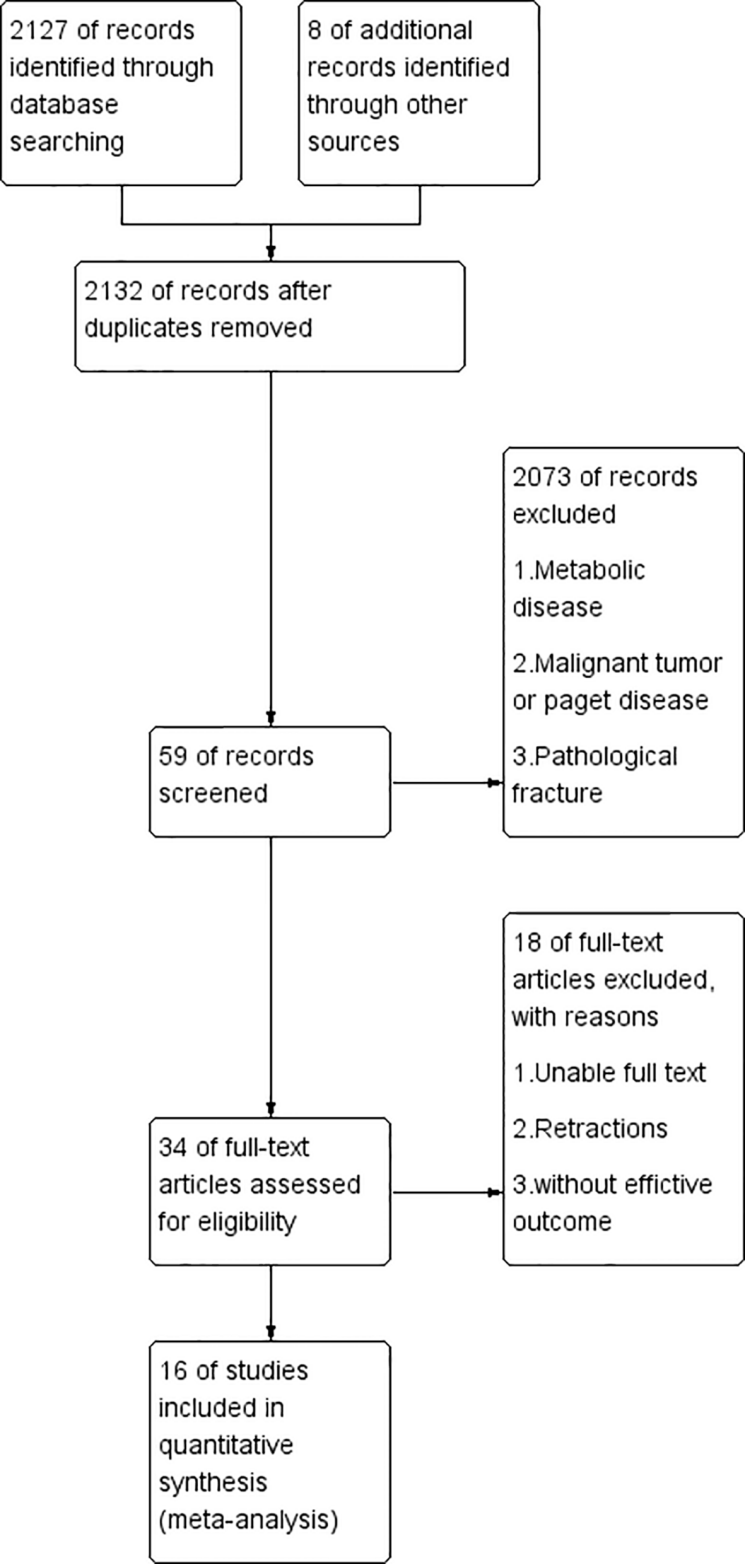
Flow diagram for the review process.

As of June 2021, a total of 2127 published articles were retrieved, in which 34 were considered potentially meaningful. By reviewing the full text, 18 articles were abandoned for the following reasons: absent of useful outcome data; unclear description; tumors; joint replacement surgery; Metabolic diseases other than osteoporosis; dialysis/transplantation; Paget disease. In the end, 16 articles were included to evaluate the relationship between BPs and fracture healing. The process is outlined in **Figure 1**.

### Study Characteristics

All the studies were published from 1987 to 2021. All the characteristics of the studies were summarized in **Table 1**, including basic information of the paper (first author and year of publication), basic information of patients, detailed types of BPs, and fracture type.

**Table 1:** Characteristics of patients included studies. “-” means that it cannot be obtained from the full text.

Study	Patients(n)	Age	Female ratio	BMI (kg/m^2^)	Type of fracture	Type of treatment	bisphosphonates	Follow-up time
**Adolphson. (** **25** **)**	32	62(50-76)	100%	–	Colles	closed reduction	Clodronate	8 weeks
**Altintaş, F. (** **26** **)**	46	–	–	–	Hip Fracture	Surgery	risedronate	3 months
**Cecilia, D. (** **27** **)**	239	81 ± 7	79.8%	25.3	Hip fracture	Surgery	alendronate	1 years
**Colón-Emeric, C. S. (** **28** **)**	537	–	76.1%	24.7	Hip Fracture	Surgery	Zoledronic acid	1.9 years
**Gong, H. S. (** **29** **)**	50	>50	100%	–	Distal Radial Fractures	Surgery	several	3 months
**Harding, A. K. (** **30** **)**	46	49 (37–63)	21.7%	27.1	OA high tibial osteotomies	hemicallotasis technique	Zoledronic acid	1.5 years
**Kim, T. Y. (** **31** **)**	51	–	–	–	intertrochanteric fractures	Surgery	risedronate	1 year
**Li, C. (** **32** **)**	82	63.73 ± 6.03	64.6%	22.89	Lumbar	TLIF surgery	Zoledronic acid	1 year
**Reid, I. R. (** **33** **)**	1367	75.8	90.1%	–	Hip Fracture	Surgery	Zoledronic acid	1 year
**Rozental, T. (** **34** **)**	196	69.0	79.1%	–	Distal Radial Fractures	several	Alendronate & risedronate	1 year
**Sato, Y. (** **35** **)**	80	75(70-79)	100%	–	Hip Fracture	Surgery	Etidronate	3 months
**Shi, C. (** **36** **)**	63	77.14	46.0%	30.95	OVCF	PKP surgery	Zoledronic acid	2 years
**Uchiyama, S. (** **37** **)**	80	70.1	38%	–	Distal Radial Fractures	Surgery	alendronate	6months
**van der P. (** **38** **)**	41	46.0	43.9%	25.2	Lower Leg fracture	Surgery	Alendronate	1 year
**Zhang, J. (** **39** **)**	101	64.29	43.9%	26.1	Lumbar	Surgery	Zoledronic acid	1 year
**Duckworth, A. D. (** **40** **)**	421	63.4	86%	–	Distal Radial Fractures	several	Alendronate	6months

The patient characteristics are summarized in **Table 1**. This meta-analysis included a total of 5022 patients. Regarding the fracture types, four trials included fractures of the distal radius (25, 29, 34, 37, 40), six trials were hip fracture (26–28, 31, 33, 35), three trial were spinal fractures (32, 36, 39), one trial was lower leg fracture (38), and one trial was OA high tibial osteotomies (30). There was also variability in the type of treatment, surgical *versus* nonsurgical. There was no significant difference between cases and controls in all the included studies.

In four trials, patients received Alendronate (27, 37, 38, 40), six trials used zoledronic acid (28, 30, 32, 33, 36, 39), two trials used risedronate (26), one trial used Etidronate (35), one trial used Clodronate (25) and two trials used multiple bisphosphates (29, 34). Besides Bisphosphate, in most of study, patients also received other treatment, such as, vitamin D and calcium supplements.

### Quality of Trials

**Figure 2:**
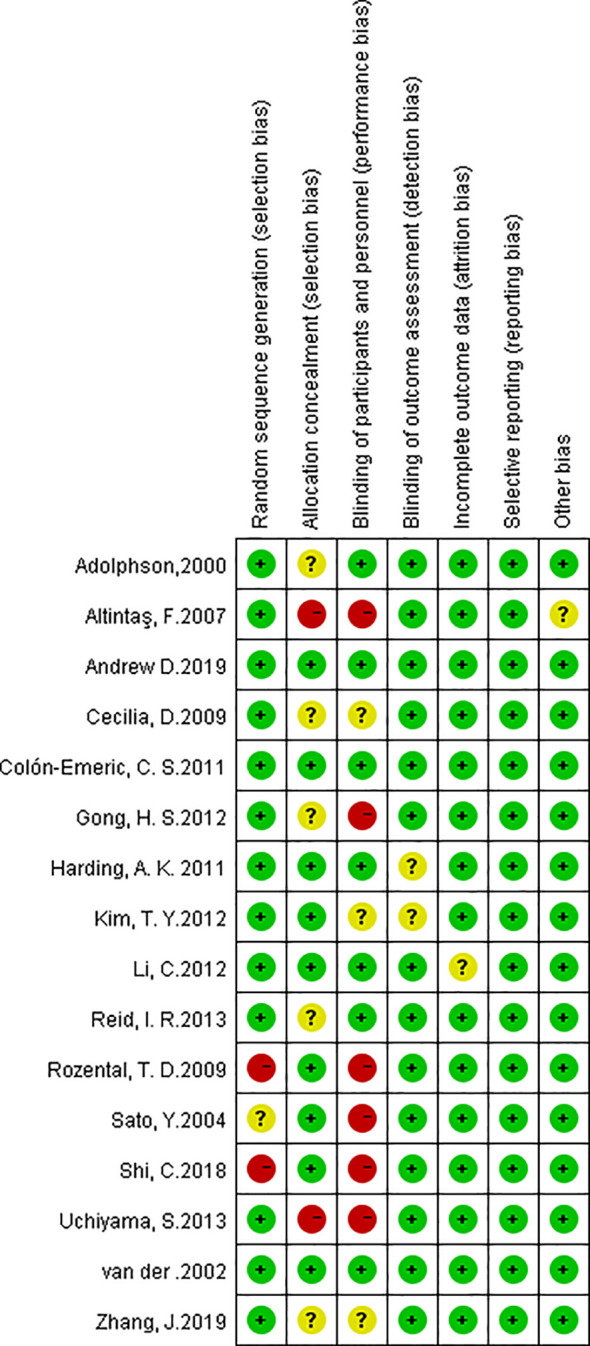
Risk of bias summary. The red with a minus means high risk of bias; the yellow with a question mark means equivocal; the green with a plus means low risk of bias.

All 16 studies were assessed by the Cochrane Scale (**Figure 2**). Green meant low risk, red meant high risk, and yellow meant unclear risk. Two studies scored 7 and most of articles scored at least 6. One article was written in Turkish, we used Google Translate (https://translate.google.cn/) to extract data and explore the study design.

### Fracture Healing Time

**Figure 3:**
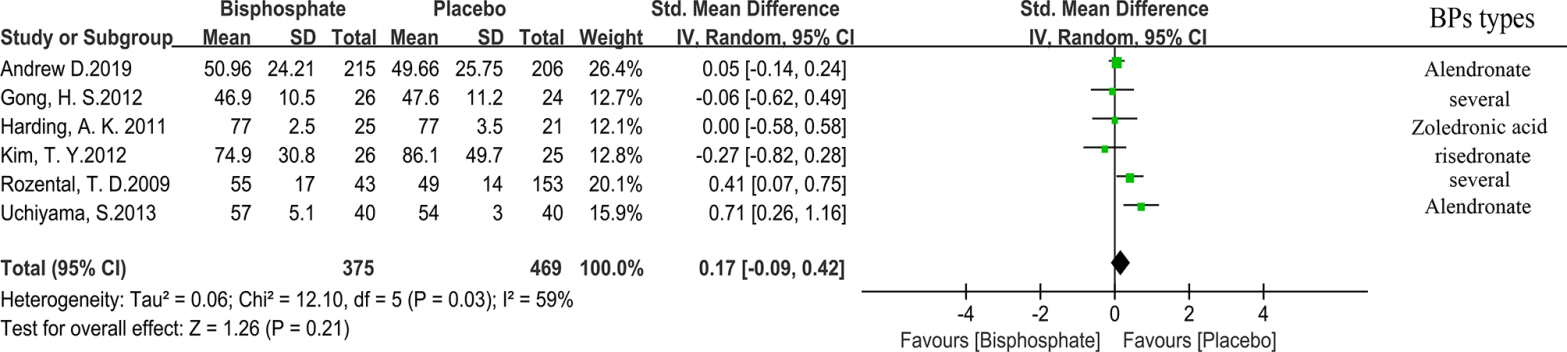
Forest Plot of Fracture Healing time.

All studies used medical imaging to determine the extent and time of fracture healing. Through expanded data analysis, we confirmed that the fracture healing time is not related to the use of BPs (**Figure 3**). 423 patients from five trials were eligible for the meta-analysis. (SMD 0.17, 95% CI −0.09 to 0.42; I^2^ = 59%, P=0.21; Radom effects model).

### Bone Mineral Density Changes

**Figure 4:**
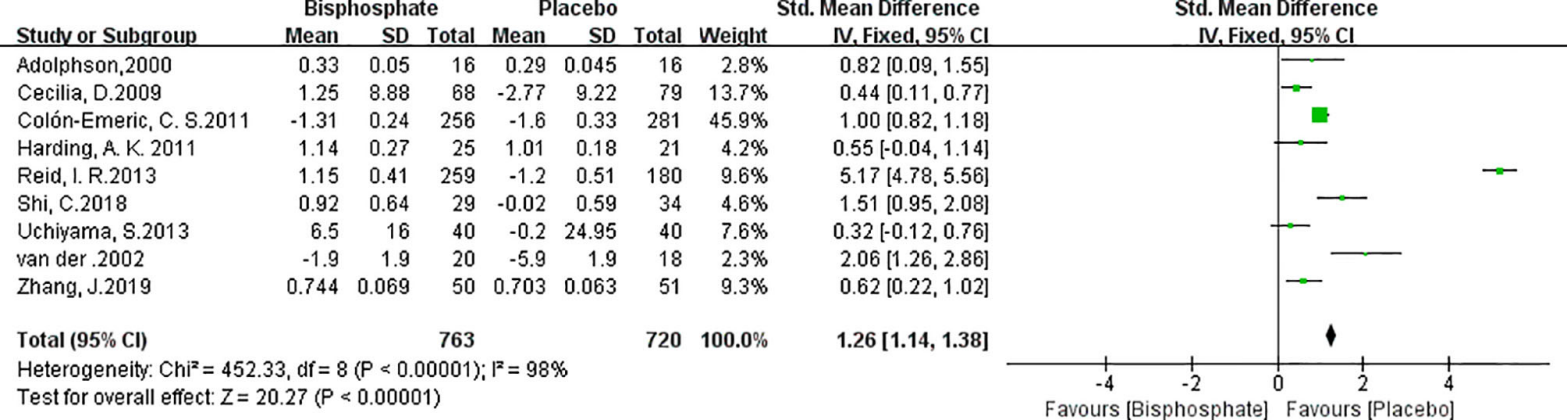
Forest Plot of BMD changes between bisphosphonate and placebo.

BMD changes reflects changes in bone metabolism which is critical for fracture healing. Unfortunately, the description of BMD and units in the various studies were not uniform and many studies lacked baseline data. In order to normalize the data, the changes in BMD are recalculated and expressed as a percentage of the initial BMD (41). Data from 9 trials (1140 patients) showed that there was a significant increase of BMD in the bisphosphonate group compared with the placebo treatment group (**Figure 4**). (SMD 2.31, 95% CI 0.38 to 2.39; I^2^ = 98%, P=0.007; Radom effects model).

In the subsequent subgroup analysis (**Figure S2**), it has been found that the trials using alendronate had higher degree of variability compared to zoledronic acid and chlorophosphate. Fracture types had no statistically significant effect on fracture healing.

### Bone Turnover Markers Assessment

Various types of bone turnover markers were used in different trials. In order to obtain sufficient and accurate data, the markers were summarized in our analysis. P1NP was used in Reid et al. (33) and Li et al. (32). ALP was used in Uchiyama et al. (29) and van der et al. (38). Sato et al. (35) used BGP. NTX was used as the bone resorption markers in all trials. CTX was used in Zhang et al. (39). Subgroup analysis found that different types of markers had no statistically significant effect on the results.

**Figure 5:**
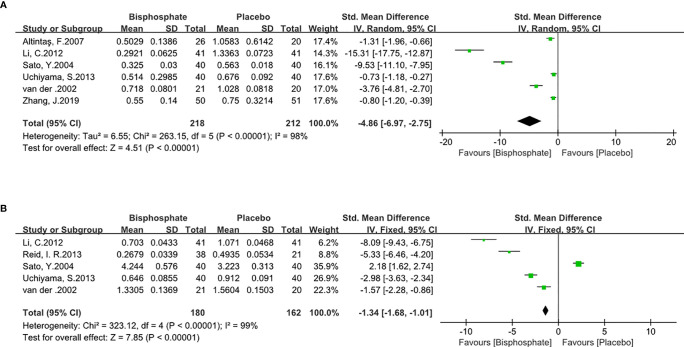
Forest Plot of Bone Turnover Markers **(A)**. Bone resorption biomarker. **(B)**. Bone formation biomarker.

BPs can significantly inhibit bone resorption (SMD -4.86, 95% CI -6.97 to 2.75; I^2^ = 98%, P<0.00001; Radom effects model) and bone formation markers (SMD -1.34, 95% CI -1.68 to -1.01; I^2^ = 98%, P<0.00001; Fixed effects model). As expected, BPs causes low bone turnover (**Figure 5**) which means BP can increase BMD.

### Sensitivity Analysis

In order to determine the contribution of individual studies, the results were pooled for sensitivity analysis. We removed each study from the analysis and determined pooled SMD. Significant change of I2 (98%→77%) and SMD (1.26→0.84) occurred in BMD change Forest plot with Reid et al. (33) deleted. Results showed BPs increased bone density. In other words, while the sensitivity of the study may have been affected, the results did not change.

### Publication Bias

**Figure 6:**
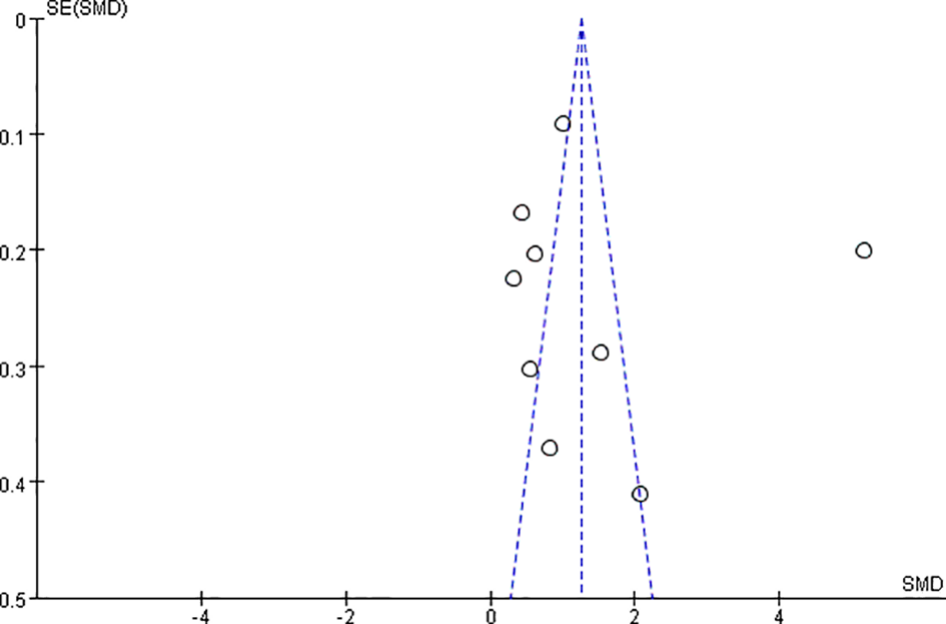
Funnel plot of BMD changes between bisphosphonate and placebo.

Publication bias was assessed by generating and analyzing a funnel plot for the analysis of BMD changes (**Figure 6**) with Reid et al. (33) deleted, the funnel plot performed more symmetrical (**Figure S1**), which also means this study may be a deviation in the meta-analysis.

## Discussion

Fracture healing depends on the dynamic balance between bone formation and bone resorption. Any factors that changes the balance can affect the fracture healing time and prognosis of the injury. BPs inhibit osteoclast activities resulting in decreased bone resorption. It is commonly used for osteoporosis and metabolic bone diseases (42–44). The BPs all have two phosphonate groups that share a common carbon atom (P-C-P), which can inhibit the synthesis of ATP or the activity of FPPS (farnesyl pyrophosphate synthase) in osteoclast and induce apoptosis. BPs can also bind to bone mineral and inhibit the dissolution of hydroxyapatite(HAP).

Since BPs inhibit bone resorption (16, 45), it may have negative effects on the bone remodeling, thereby prolonging fracture healing. One study has shown the anti-fracture efficacy of intravenous zoledronic acid in patients with recent low trauma hip fractures (46).

Long-term use of BPs can have serious adverse reactions, including osteonecrosis of the jaw and low-stress fractures (47–49). It is unclear if BPs should be used in the early stage of fracture patients (12, 39, 50, 51). Animal experiments have shown that BPs will not inhibit bone healing in rats (11, 52, 53) and it can have a positive effect on the early healing of metaphyseal fractures in mice (15). Recent study has shown that pamidronic acid has significant effect on bone mineral content and strength recovery in fractured rats (54). Early application of BPs has been shown to significantly reduce pain (55) which is conducive to early rehabilitation exercise and improves quality of life.

A clear positive effect of BPs have been found on fracture healing in animal experiment (14, 15). Our analysis found that post traumatic/postoperative application of BPs has no significant effect on fracture healing time which differs from previous studies (56) (SMD=0.17, 95% CI : −0.09 to 0.42, p=0.21). This result also indicates that BPs have no negative effect on the fracture healing in human patients, and will not lead to delayed union and nonunion. Our meta-analysis showed that compared with placebo, BPs significantly increased the BMD in patients with fractures. In the subgroup analysis, we found that alendronate has a more significant effect on BMD changes compared with zoledronate or chlorophosphite. More large number randomized control trials are needed to further elucidate the effects of BPs on fracture healing time. The use of BPs showed no discernable negative effects on fracture healing.

Significant BMD reduction and low body weight can be observed within 1 year after fracture, which can result in recurrent fractures (2). Early application of BPs can effectively increase bone density which can improve patients’ quality of life and functional recovery. Previous studies have shown that different R substituents of bisphosphonate molecules have significantly influence on binding ability to bone minerals, which also affects its clinical effect. Third-generation BPs, such as, zoledronic has better binding ability to HAP compared to alendronate (57), while zoledronic has a stronger ability to inhibit FPPS, which result to stronger inhibition to osteoclast. In other studies, the N-H-O hydrogen bond formed by the zoledronic acid and FPPS enzyme can inhibit the effect of FPPS more compared with alendronate (58). This is consistent with our analysis results. In research of spine fractures and non-spine fractures, the onset time of zoledronic acid is also shorter than that of alendronate. BPs increases callus thickness and improves VAS and disability scores (13, 20, 55, 59–62). Hence, early application of BPs can effectively increase bone density, which can improve patients` quality of life and functional recovery.

Type I collagen is the main component of bone organic matter. Type I collagen is consistent of NTX and CTX, which have a similar metabolic process. NTX and CTX are often used as bone resorption markers. P1NP, ALP, and BGP are typical osteogenic markers. Although several biomarkers are used in our research, they all have significant changes and have no impact on inference. All of the bone metabolic markers have a significant impact on changes in bone density and bone mass (63–65). The forest plot showed that bisphosphonates have a clear inhibitory effect on the bone resorption markers and bone formation markers, resulting in a low bone turnover state. BPs is, therefore, not recommended after fracture because of the resultant low bone turnover state in these studies. Other studies, however, have shown that BPs do not inhibit fracture healing (12, 17, 66).

It should be clearly stated that anatomic reduction and fixation are still the gold standard treatment for fracture healing (67). BPs, other metabolically modifying drugs, and rehabilitation are adjunct options with beneficial effects in specific situations.

There are limitations to this meta-analysis. In order to obtain exact data, which are not directly available in some manuscripts, we used software to identify the Figure from full text. Through comparison with known data, some data have proportionable certain errors (less than 5%), which may affect the results and conclusions of the analysis. The bone density was normalized which may have introduced a degree heterogeneity in the meta-analysis. The data of the included studies are not uniform. For example, NTX was used as a bone resorption marker in most of the study whereas CTX was used in others (32, 39) due to the lack of NTX data. This may introduce a certain degree of errors and biases. This is also one of the possible reasons why I^2^ was high in multiple Forest Plots. Lastly, the types of BPs used in the studies were different, which may have different clinical effects.

## Conclusion

This meta-analysis showed that bisphosphonate have no significant effect on fracture healing time but they do increase the changes in BMD and reduce bone synthesis and resorption markers. The application of bisphosphate in the early stage after injury is a correct choice for fracture healing.

## Data Availability Statement

The raw data supporting the conclusions of this article will be made available by the authors, without undue reservation.

## Author Contributions

YqG and YG reviewed the literature and extracted the data. DS, MD, and JN assessed the quality of included studies. YqG wrote the manuscript, and designed the figures and tables. XL and LL provided some key ideas for this manuscript. GH critically revised the manuscript. All authors contributed to the article and approved the submitted version.

## Funding

This study was funded by National Nature Science Foundation of China (NSCF) (Grant No: 81802207), the Xiangya Famous Doctor Fund of Central South University(Grant 2014-68), and The Hunan province Science Fund for Distinguished Young Scholars (Grant 2018JJ1046).

## Conflict of Interest

The authors declare that the research was conducted in the absence of any commercial or financial relationships that could be construed as a potential conflict of interest.

## Publisher’s Note

All claims expressed in this article are solely those of the authors and do not necessarily represent those of their affiliated organizations, or those of the publisher, the editors and the reviewers. Any product that may be evaluated in this article, or claim that may be made by its manufacturer, is not guaranteed or endorsed by the publisher.

## Supplementary Material

Supplementary Figure 1 Funnel plot of BMD changes with Reid, I. R. 2013 deleted.

Supplementary Figure 2 subsequent subgroup analysis of Fracture Healing time.
